# The Human Telomere Sequence, (TTAGGG)_4_, in the Absence and Presence of Cosolutes: A Spectroscopic Investigation

**DOI:** 10.3390/molecules19010595

**Published:** 2014-01-06

**Authors:** Vishal R. Sharma, Richard D. Sheardy

**Affiliations:** Department of Chemistry and Biochemistry, Texas Woman’s University, Denton, TX 76204, USA; E-Mail: vsharma@twu.edu

**Keywords:** DNA quadruplexes, circular dichroism, PEG, osmotic stress

## Abstract

Historically, biophysical studies of nucleic acids have been carried out under near ideal conditions, *i.e*., low buffer concentration (e.g., 10 mM phosphate), pH 7, low ionic strength (e.g., 100 mM) and, for optical studies, low concentrations of DNA (e.g., 1 × 10^−6^ M). Although valuable structural and thermodynamic data have come out of these studies, the conditions, for the most, part, are inadequate to simulate realistic cellular conditions. The increasing interest in studying biomolecules under more cellular-like conditions prompted us to investigate the effect of osmotic stress on the structural and thermodynamic properties of DNA oligomers containing the human telomere sequence (TTAGGG). Here, we report the characterization of (TTAGGG)_4_ in potassium phosphate buffer with increasing percent PEG (polyethylene glycol) or acetonitrile. In general, the presence of these cosolutes induces a conformational change from a unimolecular hybrid structure to a multimolecular parallel stranded structure. Hence, the structural change is accompanied with a change in the molecularity of quadruplex formation.

## 1. Introduction

Most of us think of DNA as the right handed double helical structure as proposed by Watson and Crick [1] in the early fifties. However, with the discovery of left handed DNA in 1979 [2] came the realization that DNA can assume different structures or, more correctly, different conformations. It is now recognized that DNA can assume many different conformations: single stranded, double stranded, triple stranded and multi stranded. Further, double helical conformations can either be right handed or left handed. However, the conformation that DNA can assume *in vitro* depends highly on the sequence of the bases (the so called sequence context) as well as the conditions (e.g., temperature, pH, cations present and their concentrations) under which the DNA is prepared.

We know that the sequence of the bases within a gene contains the genetic information (*i.e*., the genetic code) as well as information for the self-processing of the DNA. It is also now clear that the sequence of the bases determine the conformation of a segment of the DNA as well. The questions, then, are what *in vivo* DNA conformations are biologically relevant and what is the relationship between conformation and function? Of particular interest recently are the conformations available to G-rich DNAs which are designated as G-quadruplexes. DNA sequences that have islands of G_2–4_ separated by 1 to 4 A and/or T bases can form a rich library of secondary structures with different molecularities, strand orientations, and guanine base conformation (*i.e*., *syn* or *anti*) [3,4,5,6,7,8,9,10,11,12,13,14]. The conformations and their stabilities are highly dependent upon the sequence context and buffer conditions [15,16,17,18,19,20,21].

We have previously reported structural and thermodynamic studies on the human telomeric repeat (TTAGGG)_4_ in the presence of potassium ion [19]. Circular dichroism (CD) studies indicated the presence of a single intramolecular hybrid quadruplex prior to heat induced unfolding. A model was presented for the quadruplex to random coil transition, via formation of an unknown intramolecular intermediate, with exclusion of K^+^ during each transition. Review of the literature reveals that different investigators have used slight variations of the sequence (TTAGGG)_4_ for their investigations and have drawn conclusions based upon their data [3,4,5,6,7,8,9,10,11,12,13,14,15,16,17,18,19,20,21]. We recently reported however that changing just one base in just one loop of the folded quadruplex influences both conformation and stability [20].

Conformational and thermal stability studies have historically been carried out under near ideal conditions of low buffer concentrations with low ionic strength and DNA concentrations on the order of 10^−4^ to 10^−6^ M. Although valuable data have come out of these studies, the solution conditions, for the most part, are inadequate to simulate realistic cellular conditions. To address this issue, carrying out these biophysical characterizations in the presence of molecular “crowding agents” has gained popularity [22,23,24,25,26,27,28,29]. One of the earliest studies of DNA quadruplexes using molecular crowding agents was reported by Miyoshi *et al*. in 2002 [22]. They reported that increasing concentrations of PEG 200 induces a conformational transition in the sequence G_4_T_4_G_4_ from a bimolecular anti parallel quadruplex to a tetramolecular parallel quadruplex and the transition is complete by 40% PEG 200. In 2007, Xue *et al*., reported the complete conversion at 40% PEG 200 to a parallel stranded structure from a unimolecular parallel/antiparallel quadruplex for the human telomere sequence G_3_(T_2_AG_3_)_3_ [23]. Heddi and Phan investigated the effect of several crowding agents on the conformation of DNA sequences containing a G_3_(TTAG_3_)_3_ core using both CD and NMR approaches. They found that the percentage of PEG 200 required to induce a high order structure depended upon the particular sequence context of the bases flanking the G_3_(TTAG_3_)_3_ core [24]. While other studies have used different quadruplex forming DNA sequences, most have used 40% PEG 200 as the conformational transition inducer [25,26,27,28,29].

As noted above, there are many publications on the use of high molecular weight PEGs as a molecular crowding agent. The influence that PEG has on the biophysical properties of the DNA is a function of both its molecular weight and concentration. Further, the effects have been deconstructed to a preferential interaction term and an excluded volume term: preferential interactions are favored for small PEGs (MW < 440) while excluded volume effects are noted for large PEGs, at least for duplexes and hairpins [30]. Thus, PEG 200 falls under the regime of preferential interaction rather than excluded volume. Further, a recent report suggests that PEG preferentially interacts with a particular quadruplex conformation and that excluded volume effects are negligible [31]. Although the presence of PEG in solution clearly influences the conformation and stability of a DNA quadruplex, referring to it as a crowding agent is not recommended [31,32,33,34,35].

The effect of osmotic stress on the conformations and stabilities of DNA structures has also been investigated (for example, [36,37,38,39]). The use of small osmolytes to alter the activity of water reveals the role that water plays in nucleic acid structure and stability. Small neutral molecules such as betaine, sucrose, trifluoroethanol and acetonitrile are good candidates for these studies since they do not interact with the nucleic acid itself.

The biophysical characterization of G quadruplexes has received much attention due to the potential to design new therapies based on the specific molecular recognition of the DNA quadruplexes by small molecules [40,41,42,43,44]. As we, and others, have described earlier, the solution structures of DNA quadruplexes are very sensitive to changes in DNA sequence context as well as to changes in solution environment. Further, many structural and thermodynamic characterizations have typically been carried out in close to ideal solutions, e.g., 10 mM phosphate buffer, 115 mM K^+^, pH 7.0. In addition, optical characterizations by UV/Vis or CD spectroscopies mandate dilute solutions (10^−4^ to 10^−6^ M) of the DNA. In this study, we have carried out CD spectroscopic studies of the human telomere sequence (TTAGGG)_4_ under different solution conditions in the absence or presence of cosolutes PEG and acetonitrile to assess the effect of osmotic stress on the conformation and stability of the quadruplex formed. As expected, the conformation of the quadruplex and its stability is exquisitely sensitive to environmental conditions.

## 2. Results and Discussion

**Figure 1:**
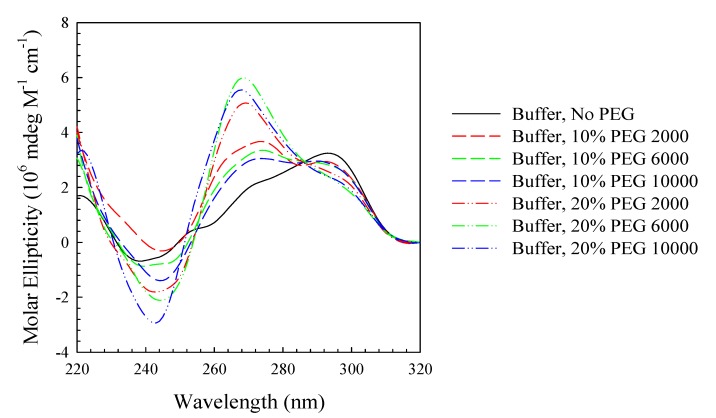
CD spectra at 25 °C in standard potassium phosphate buffer (115 mM K^+^) of (TTAGGG)_4_, in the absence (solid black line) or presence of 10% (dash) or 20% (dash-dot-dot) PEG 2,000 (red), PEG 6,000 (green) or PEG 10,000 (blue).

Figure 1 displays the CD spectra of (TTAGGG)_4_ in standard phosphate buffer with 115 mM K^+^ in the presence or absence of PEG of different molecular weights and percentages. The CD spectrum of (TTAGGG)_4_ in the absence of PEG is similar to those previously published and is consistent with a unimolecular hybrid structure with two lateral loops and one propeller loop [19,20,21]. This spectrum is characterized by a trough at 242 nm, a peak at 292 nm and a shoulder at 272 nm. The presence of 10% PEG shifts the peak intensities such that the major peak is found at 272 nm and the shoulder is found at 292 nm. As the percentage of PEG increases to 20%, the shoulder at 292 nm gradually fades away and the peak at 272 nm intensifies. These spectra have characteristics similar to a fully parallel stranded structure [45]. Further, these spectra are strikingly similar to those reported by us for DNA oligomers of sequence (CCCCTTTTGGGGT_1–4_GGGG ) which self-assemble into high molecular weight species of multiple molecularties in the presence of Mg^2+^ [46,47].

**Figure 2:**
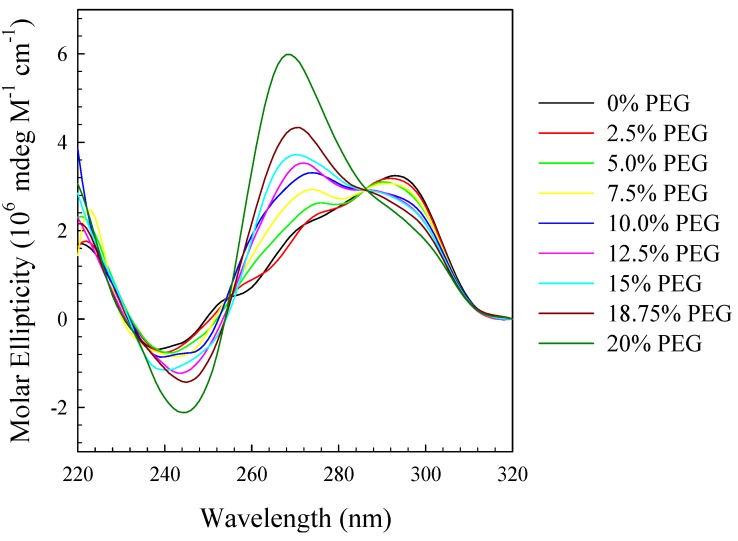
CD spectra at 25 °C in standard potassium phosphate buffer (115 mM K^+^) of (TTAGGG)_4_ in the absence or presence of increasing weight percent PEG 6,000 as indicated on the figure.

Figure 2 displays the CD spectra at 25 °C of (TTAGGG)_4_ in standard phosphate buffer, 115 mM K^+^, with PEG 6,000 ranging from 0% to 20%. As can be seen, there is a gradual transition from the normal CD spectrum to one which is very similar to that observed for a parallel stranded conformation. The peak at 292 nm slowly diminishes while the shoulder at 272 nm develops into a very intense peak at 268 nm. Further, there is a well-defined isoelliptic point at 286 nm characteristic of a two state transition. As noted above, the CD spectrum at 20% PEG 6,000 is very similar to those reported earlier for the self-assembled, multimeric species formed from (CCCCTTTTGGGGT_1–4_GGGG) [46,47].

**Scheme 1:**
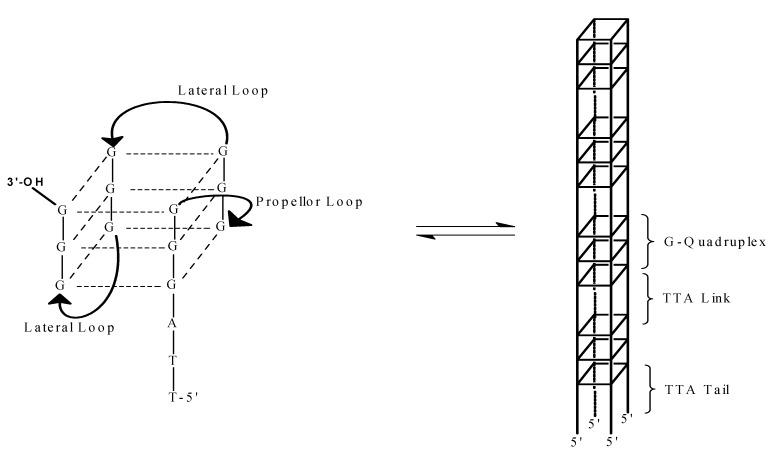
The transition for a unimolecular conformation to a termolecular conformation. The depiction on the left is for the unimolecular conformation possessing a propeller loop and two lateral loops. This conforms with the structure of (TTAGGG)_4_ in the presence of K^+^ [8]. The depiction on the right is a schematic for the parallel stranded conformation with molecularity of four. Here, three layered G-tetrads are joined by –TTA- linkers.

These data suggest the following equilibrium:

Conformer I ⇌ Conformer II

where Conformer I is the hybrid form depicted schematically on the left in Scheme 1 and Conformer II is depicted schematically on the right in Scheme 1. Thus PEG is driving the equilibrium to the side of Conformer II with increasing concentrations as observed before with different G-rich sequences [22,23,24,25,26,27,28,29,31]. According to Chaires *et al*., this shift in equilibrium is due to conformational selection rather than excluded volume or crowding effects [31]. None the less, the high concentrations of PEG will slightly influence the activity of water which will contribute to overall conformational stability.

**Figure 3:**
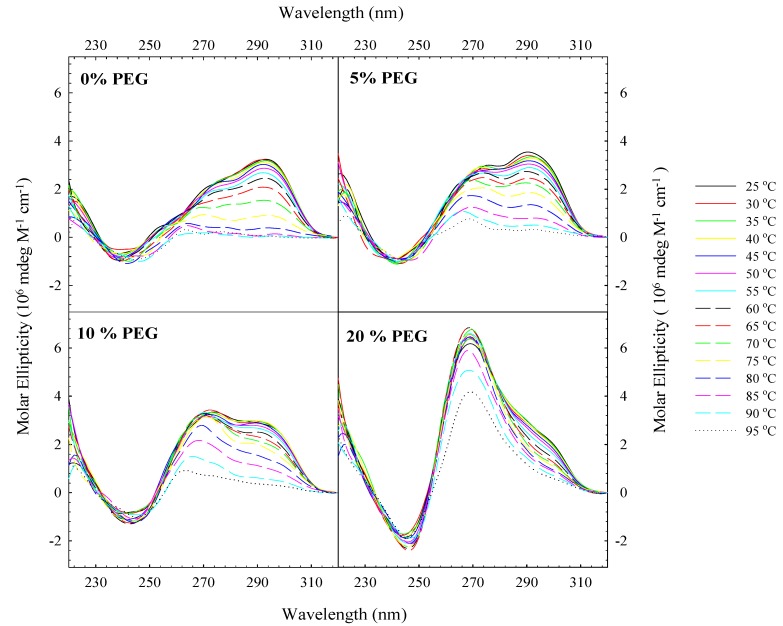
Typical CD optical melting spectra of (TTAGGG)_4_ in standard potassium phosphate buffer (115 mM K^+^) as a function of percent weight PEG 6,000.

CD optical melting studies were carried out for (TTAGGG)_4_ as a function of % PEG 6,000. Figure 3 shows CD spectra for (TTAGGG)_4_ as a function of temperature with different % PEG 6,000. In the absence of any PEG, (TTAGGG)_4_ undergoes a transition from the fully folded state at 25 °C to the fully unfolded state at 95 °C as previously reported [19]. The spectra recorded at 85 °C, 90 °C and 95 °C are nearly superimposable indicating that the oligomer is fully unfolded by 85 °C. However, the 90 °C and 95 °C spectra are not superimposable in the presence of 5% PEG suggesting that the oligomer may not be fully unfolded at 95 °C. Increasing the percent of PEG further leads to even more incomplete unfolding at 95 °C as can be seen for the 10% and 20% PEG spectra.

**Figure 4:**
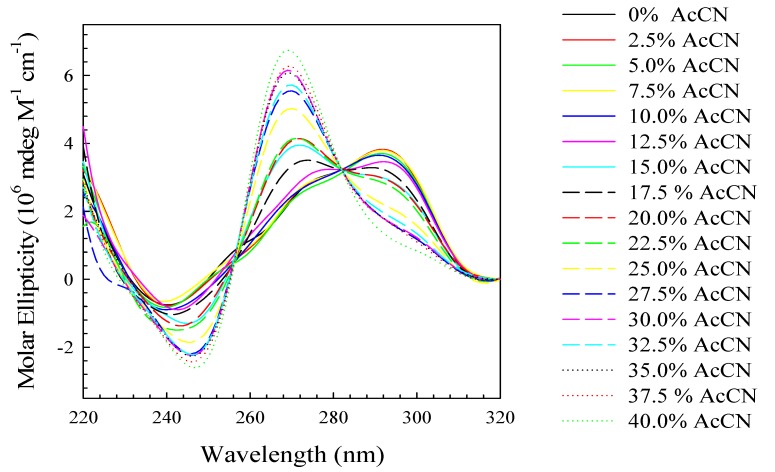
CD spectra at 25 °C in standard potassium phosphate buffer (115 mM K^+^) of (TTAGGG)_4_ in the absence or presence of increasing percent acetonitrile (AcCN) as indicated on the figure.

To investigate the activity of water on the conformation of (TTAGGG)_4_, we investigated the influence of acetonitrile—a known water disrupter. As can be seen in Figure 4, increasing the concentration of acetonitrile in the buffer system also induces a conformational change to a structure whose CD spectrum is similar to a parallel stranded quadruplex. The observation of an isoelliptic point at 282 nm is also consistent with a two state transition. None the less, this conformational transition is strictly due to the dehydration effects of acetonitrile.

**Figure 5:**
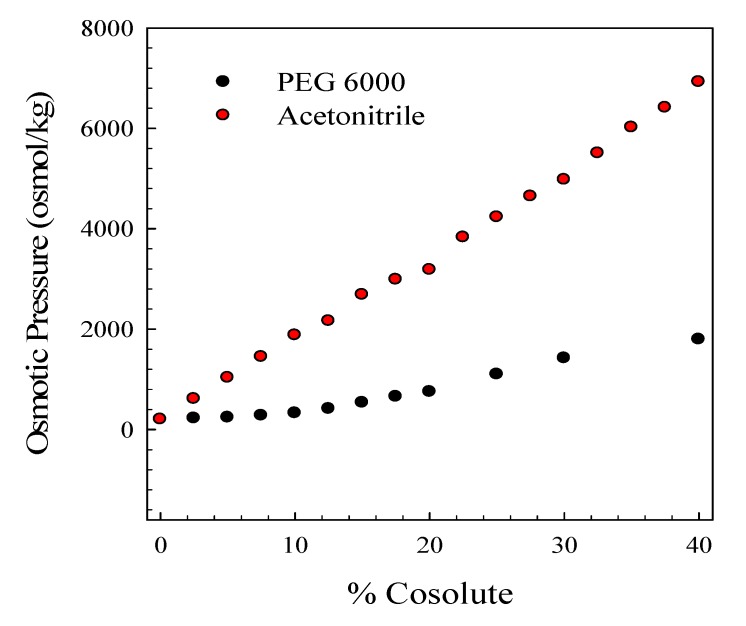
A comparison of the percent cosolute to osmotic pressure for acetonitrile (% V/V) and PEG 6,000 (% w/V) in standard phosphate buffer, pH 7.0, 115 mM K^+^.

We compared the osmolality of the buffers solutions used for these studies. A plot of milliosmolality *vs*. % cosolute (PEG 6,000 *vs*. acetontirile) is shown in Figure 5. Since osmolality is directly proportional to ln (α_W_), where α_W_ is the activity of water, acetonitrile is clearly more effective in disrupting water activity than is PEG 6,000. It is also interesting to compare the linear behavior of acetonitrile to the non-linear behavior of PEG. This deviation from linearity may be due to a number of factors including association with the DNA conformation as well as self-aggregation.

**Figure 6:**
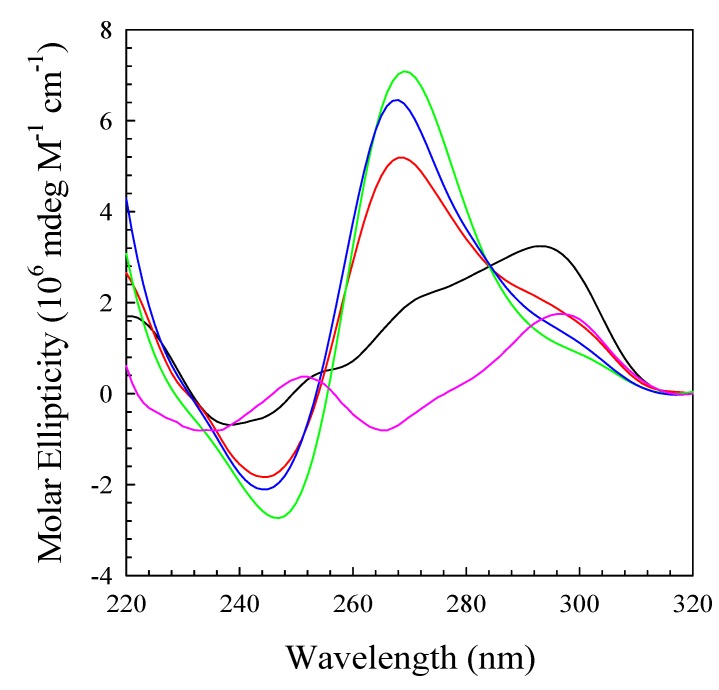
CD spectra of (TTAGGG)_4_ under five different conditions: (1) 10 mM phosphate, pH 7.0, 115 mM Na^+^ (pink); (2) 10 mM phosphate, pH 7.0, 115 mM K^+^ (black); (3) 10 mM phosphate, pH 7.0, 115 mM K^+^, 20% PEG 6000 (red); (4) 10 mM phosphate, pH 7.0, 115 mM K^+^, 40% acetonitrile (green); and (5) 10 mM TBE buffer, pH 8.0, 115 mM K^+^, 20 mM Mg^2+^ (blue).

**Scheme 2:**
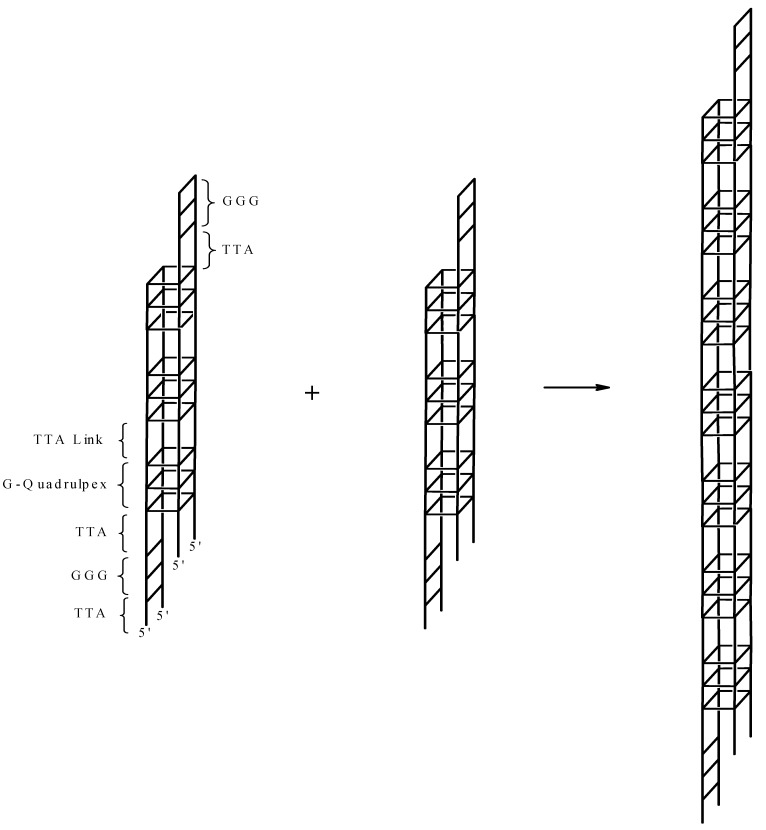
The self-assembly of (TTAGGG)_4_.

The CD spectra of (TTAGGG)_4_ in 20% PEG or 40% acetonitrile suggests that the conformation under these conditions is that of a parallel stranded quadruplex as previously suggested [23]. As noted above, one possibility for this structure is depicted on the right panel of Scheme 1. Previously, we reported the self-assembly of DNA oligomers of general sequence C_4_T_4_G_4_T_1-4_G_4_ into high molecular weight species in the presence of Mg^2+^ [46,47]. In the absence of Mg^2+^, the oligomers did not self-assemble and behaved as hairpin conformations. Evidence for this self-assembly came from both CD data and gel electrophoresis studies. For the CD and electrophoreses studies, the oligomers of interest were prepared in 10 mM TBE, pH 8.0, 115 mM K^+^ and 20 mM Mg^2+^. Thus, Mg^2+^ stabilizes the multimeric parallel stranded conformation to a greater extent than the unimolecular hairpin. Figure 6 is a comparison of the CD spectra of (TTAGGG)_4_ under five different conditions: (1) phosphate buffer, pH 7.0, with 115 mM Na^+^; (2) phosphate buffer, pH 7.0 with 115 mM K^+^; (3) phosphate buffer, pH 7.0 with 115 mM K^+^; and 20% PEG 6000; (4) phosphate buffer, pH 7.0 with 115 mM K^+^ with 40% acetonitrile; and (5) 10 mM TBE, pH 8.0, 115 mM K^+^ and 20 mM Mg^+^. Note that the spectra determined in the presence of PEG, acetonitrile and TBE-Mg^2+^ are very similar indicating the conformation for the DNA oligomer is the same under all three conditions. Since we know that Mg^2+^ drives the formation of a self-assembled system, there is no reason to think that (TTAGGG)_4_ does not self-assemble in PEG or acetonitrile as depicted in Scheme 2.

The CD data suggest that the folded quadruplex undergoes a transition from the hybrid unimolecular conformation to a parallel stranded conformation, most likely a conformation with the molecularity of at least four, in the presence of PEG, acetonitrile or Mg^2+^ as depicted in Scheme 1. Hence, the molecularity of the quadruplex increases from one to at least four. In consideration of these conformational and stability studies, another possibility must be considered for the structure of the quadruplex formed from (TTAGGG)_4_ under these conditions. The CD and melting data for this oligomer is strikingly similar to the data observed for the self-assembly of (CCCCTTTTGGGGT_1–4_GGGG) [46,47]. The conformation shown in Scheme 1 for the parallel stranded structure assumes that all four strands are in “register”—the first G triplets in each strand form the first G quadruplex in the four stranded structure. Consider the possibility of two strands out of register as shown in the far left schematic in Scheme 2. This would create a four stranded structure with “sticky” ends that could self-assemble into very high molecular weight structures as depicted in the far right schematic. Hence, the formation of so called “G wires” cannot be ruled out [48,49,50]. We are currently investigating this possibility.

## 3. Experimental Section

### 3.1. Preparation of Buffer

Standard potassium phosphate buffer (10 mM phosphate, 0.1 mM EDTA, 15 mM K^+^, pH 7.0) was prepared using KH_2_PO_4_ (VWR International, Sugar Land, TX 77478), K_2_HPO_4_ (VWR International), and EDTA (EMD Chemicals, Darmstadt, Germany). KCl (Thermo Fisher Scientific, Waltham, MA 02454, USA) was added to give a final concentration 115 mM K^+^. Polyethylene glycol (Alfa Aesar, Ward Hill, MA 01835, USA) of molecular weights 2,000, 6,000, or 10,000 or acetonitrile was added to the standard potassium phosphate buffer to make various solutions of different percent cosolute. Standard sodium phosphate buffer (10 mM phosphate, 0.1 mM EDTA, 15 mM Na^+^, pH 7.0) was prepared using NaH_2_PO_4_ (Fisher Scientific), Na_2_HPO_4_ (Fisher Scientific) and EDTA (EMD Chemicals). NaCl (Fisher Scientific) was added to give a final concentration of 115 mM Na^+^. TBE buffer (10 mM TRIS-borate, pH 8.0, 115 mM K^+^ and 20 mM Mg^2+^) buffer was prepared using Trizma Base (Sigma Aldrich, St. Louis, MO 63103, USA), boric acid (Fisher Scientific), KCl and MgCl_2_ (Fisher Scientific). All buffer solutions were filtered through a 0.45 μm Millipore filter and degassed before being stored for use.

### 3.2. DNA Oligomers

HPLC purified (TTAGGG)_4_ was purchased from Biosynthesis, Inc. (Lewisville, TX, USA) and used without further purification. All sequences were reconstituted in about 1 mL of buffer, heated to 95 °C, slowly cooled to room temperature and stored at 4 °C. The extinction coefficient, provided by the supplier, was 244,600 M^−1^ cm^−1^ in bases for (TTAGGG)_4_ at 260 nm.

### 3.3. UV-Vis Spectroscopy

A Varian Cary 100 Bio model (Varian Associates, Palo Alto, CA, USA) UV/Vis spectrometer was used to measure the concentration of the samples used in circular dichroism. Appropriate dilutions of each CD sample were made and run in 10 mm square quartz cuvettes from 320–220 nm at 25 and 95 °C with the appropriate baseline subtracted. Concentrations were determined using the extinction coefficient above and are reported in bases/liter.

### 3.4. Circular Dichroism

All circular dichroism studies were carried out using an Olis RSM 1,000 CD spectrophotometer (Olis Inc., Athens, GA, USA). Data were collected over the range of 320 nm to 220 nm at temperatures ranging from 25 to 95 °C with 5 °C increments. All samples including baseline were run in 1 mm circular quartz cuvettes. Olis Global works and Sigma Plot version 11 were used to process and analyze the data. DNA concentrations for these optical studies typically range from 1 × 10^−5^ to 1 × 10^−6^ M.

### 3.5. Osmotic Pressure Determinations

Osmotic pressures of all phosphate buffer solutions in the absence or presence of PEG or acetonitrile were determined using a Model 3,320 Micro Osmometer (Advanced Instruments, Inc., Norwood, MA, USA). Osmotic pressures in mosm/kg were recorded in triplicate at 25 °C and averaged.

## 4. Conclusions

The conformational properties of G-quadruplexes are exquisitely sensitive to sequence context and environmental influences. This project focused on the affect that environmental conditions would have on the conformation and stability of the quadruplex formed from (TTAGGG)_4_, the human telomere sequence. In standard phosphate buffer with 115 mM K^+^, this sequence assumes a hybrid unimolecular conformation; in standard phosphate buffer with 115 mM Na^+^, this sequence assumes an antiparallel unimolecular conformation. As the percent of PEG or acetonitrile in standard potassium phosphate buffer is increased incrementally, a gradual change in the conformation of the quadruplex is observed to a conformation with CD spectral characteristics of a parallel stranded structure with a molecularity of at least four. The resultant CD spectra are similar to that of the sequence in the presence of Mg^2+^—a known inducer of G-wire formation. In addition, the melting temperature of the DNA quadruplex increases as the % PEG increases. In the case of acetonitrile, the conformational changes are due to changes in water activity and how water interacts with the DNA backbone and bases. The interaction of water with the backbone and bases is also affected by the binding of Mg^+^ to the DNA backbone. As in the case reported by Chaires *et al.*, the presence of PEG most likely influences the conformation and stability of the quadruplex through conformational selection of the parallel stranded structure, although disruption of water may contribute but to a lesser extent than that of acetonitrile. We are currently investigating the influence of other osmolytes and large molecules on the conformation and stability of this DNA sequence.

## References

[1] Watson J.D., Crick F.H.C. (1953). Molecular structure of nucleic acids—A structure for deoxyribose nucleic acid. Nature.

[2] Pohl F.M., Jovin T.M. (1972). Salt-induced co-operative conformational change of synthetic DNA: Equilibrium and kinetic studies with poly(dG-dC). J. Mol. Biol..

[3] Williamson J.R. (1994). G-Tetrad structures in telomeric DNA. Ann. Rev. Biophys. Biomol. Struct..

[4] Venczel E.A., Sen D. (1993). Parallel and antiparallel G-DNA structures from a complex telomeric sequence. Biochemistry.

[5] Burge S., Parkinson G.N., Hazel P., Todd A.K., Neidle S. (2006). Quadruplex DNA: Sequence, topology and structure. Nucleic Acid. Res..

[6] Wang Y., Patel D.J. (1993). Solution structure of the human telomeric repeat d[AG_3_(T_2_AG_3_)_3_] G-tetraplex. Structure.

[7] Ambrus A., Chen D., Dai J., Bialis T., Jones R., Yang D. (2006). Human telomeric sequence forms a hybrid-type intramolecular G-quadruplex with mixed parallel/antiparallel strands in potassium solution. Nucleic Acid. Res..

[8] Luu K.N., Phan A.T., Kuryavyi V., Lacroix L., Patel D. (2006). Structure of the human telomere in K+solution: An intramolecular (3 + 1) G-quadruplex scaffold. J. Am. Chem. Soc..

[9] Xu Y., Noguchi Y., Sugiyama H. (2006). The new models of the human telomere d [AGGG(TTAGGG)_3_] in K^+^ solution. Bio-Org. Med. Chem..

[10] Phan A.T., Kuryavyi V., Luu K.N., Patel D.J. (2007). Structure of two intramolecular G-quadruplexes formed by the natural human telomere sequence in K^+^ solution. Nucleic Acid. Res..

[11] Lim K.W., Amrane S., Bouaziz S., Xu W., Mu Y., Patel D.J., Luu K.N., Kim N., Phan A.T. (2009). Structure of the human telomere in K^+^ solution: A stable basket-type G-quadruplex with only two G-tetrad layers. J. Am. Chem. Soc..

[12] Kuryavyi V., Patel D. (2010). Solution structure of a unique G-quadruplex scaffold adopted by a guanosine-rich human intronic sequence. Structure.

[13] Zhang Z., Dai J., Veliath E., Jones R.A., Yang D. (2010). Structure of a two-G-tetrad intramolecular G-quadruplex formed by a variant human telomeric sequence in K^+^ solution. Nucleic Acid. Res..

[14] Viglasky V., Bauer L., Tluckova K. (2010). Structural features of intra- and intermolecular G-quadruplexes derived from the human telomere. Biochemistry.

[15] Rachwal P.A., Fox K.R. (2007). Quadruplex melting. Methods.

[16] Lane A.N., Chaires J.B., Gray R.D., Trent J.O. (2008). Stability and kinetics of G-quadruplex structures. Nucleic Acid. Res..

[17] Balkwill G.D., Garner T.P., Searle M.S. (2009). Folding of single-stranded DNA quadruplexes containing an autonomously stable mini-hairpin loop. Mol. BioSyst..

[18] Olsen C.M., Marky L. (2010). Monitoring the temperature unfolding of G-quadruplexes by UV and circular dichroism spectroscopies and calorimetry techniques. Methods Mol. Biol..

[19] Antonacci C., Chaires J.B., Sheardy R.D. (2007). Biophysical characterization of the human telomeric repeat (TTAGGG)_4_ in potassium solution. Biochemistry.

[20] Yadav D., Sheardy R.D. (2012). A single base permutation in any loop of a folded intramolecular quadruplex influences its structure and stability. J. Biophys. Chem..

[21] Tucker B.A., Gabriel S., Sheardy R.D., Sheardy R.D., Winkle S.A. (2011). A CD Spectroscopic Investigation of Inter- and Intramolecular DNA Quadruplexes. Frontiers in Nucleic Acids.

[22] Miyoshi D., Nakao A., Sugimoto N. (2002). Molecular crowding regulates the structural switch of the DNA G-quadruplex. Biochemistry.

[23] Xue Y., Kan Z.-Y., Wang Q., Yao Y., Liu J., Hao Y.-H., Tan Z. (2007). Human telomeric DNA forms parallel-stranded intramolecular G-quadruplex in K^+^ solution under molecular crowding condition. J. Am. Chem. Soc..

[24] Petraccone L., Malafronte A., Amato J., Giancola C. (2012). G-Quadruplexes from human telomeric DNA: How many conformations in PEG containing solutions?. J. Phys. Chem..

[25] Zheng K.-W., Chen Z., Hao Y.-H., Tan Z. (2009). Molecular crowding creates an essential environment for the formation of stable G-quadruplexes in long double-stranded DNA. Nucleic Acid. Res..

[26] Zhang D.-H., Fujimoto T., Saxena S., Yu H.-Q., Miyoshi D., Sugimoto N. (2010). Monomorphic RNA G-quadruplex and polymorphic DNA G-quadruplex structures responding to cellular environmental factors. Biochemistry.

[27] Heddi B., Phan A.T. (2011). Structure of human telomeric DNA in crowded solution. J. Am. Chem. Soc..

[28] Fujimoto T., Nakano S.-I., Sugimoto N., Miyoshi D. (2012). Thermodynamics-hydration relationships within loops that affect G-quadruplexes under molecular crowding conditions. J. Phys. Chem..

[29] Yu H., Gu X., Nakano S.-I., Miyoshi D., Sugimoto N. (2012). Beads-on-a-string structure of long telomeric DNAs under molecular crowding condition. J. Am. Chem. Soc..

[30] Knowles D.B., LaCroix A.S., Deines N.F., Shkel I., Record M.T. (2011). Separation of preferential interaction and excluded volume effects on DNA duplex and hairpin stability. Proc. Natl. Acad. Sci. USA.

[31] Buscaglia R., Miller M.C., Dean W.L., Gray R.D., Lane A.N., Trent J.O., Chaires J.B. (2013). Polyethylene glycol binding alters human telomere G-quadruplex structure by conformational selection. Nucleic Acids Res..

[32] Zhou H.-X., Rivas G., Minton A.P. (2008). Macromolecular crowding and confinement: Biochemical, biophysical, and potential physiological consequences. Ann. Rev. Biophys..

[33] Elcock A.H. (2010). Models of macromolecular crowding effects and the need for quantitative comparisons with experiments. Curr. Opin. Struct. Biol..

[34] Hirano A., Shiraki K., Arakawa T. (2012). Polyethylene glycol behaves like weak organic solvent. Biopolymers.

[35] Petraconne L., Pagano B., Giancola C. (2012). Studying the effect of crowding and dehydration on DNA G-quadruplexes. Methods.

[36] Nakano S.-I., Yamaguchi D., Tateishi-Karimata H., Miyoshi D., Sugimoto N. (2012). Hydration changes upon DNA folding studied by osmotic stress experiments. Biophys. J..

[37] Ruggiero N.J., Pereira De S.F., Colombo M.F. (2001). Hydration effects on DNA double helix stability modulates ligand binding to natural DNA in response to changes in water activity. Cell Mol. Biol..

[38] Priesler R.S., Chen H.H., Colombo M.F., Choe Y., Short B.J., Rau D.C. (1995). The B form to Z form transition of poly(dG-m5dC) is sensitive to neutral solutes through osmotic stress. Biochemistry.

[39] Miller M.C., Buscaglia R., Chaires J.B., Lane A.N., Trent J.O. (2010). Hydration is a major determinant of the G-quadruplex stability and conformation of the human telomere 3’sequence of d(AG_3_(TTAG_3_)_3_). J. Am. Chem. Soc..

[40] Balasubramanian S., Hurley L.H., Neidle S. (2011). Targeting G-quadruplexes in gene promoters: A novel anticancer strategy?. Nat. Rev. Drug Dis..

[41] Duchler M. (2012). G-quadruplexes: Targets and tools in anticancer drug design. J. Drug Target..

[42] Bidzinska J., Cimino-Reale G., Zaffaroni N., Folini M. (2012). G-quadruplex structures in the human genome as novel therapeutic targets. Molecules.

[43] Miller K.M., Rodriguez R. (2011). G-quadruplexes: Selective DNA targeting for cancer therapeutics?. Expert Rev. Clin. Pharmacol..

[44] Wong H.M., Payet L., Huppert J.L. (2009). Function and targeting of G-quadruplexes. Curr. Opin. Mol. Ther..

[45] Balagurumoorthy P., Brahmachari S.K., Mohanty D., Bansal M., Sassisekharan V. (1992). Hairpin and parallel quartet structures for telomeric sequences. Nucleic Acids Res..

[46] Marotta S.P., Tamburri P.A., Sheardy R.D. (1996). Sequence and environmental effects on the self-assembly of DNA oligomers possessing G_x_T_2_G_y_ segments. Biochemistry.

[47] Dai T.-Y., Marotta S.P., Sheardy R.D. (1995). Self-assembly of DNA oligomers into high molecular weight species. Biochemistry.

[48] Sen D., Gilbert W. (1992). Superstructures formed by telomere like oligomers. Biochemistry.

[49] Marsh T.C., Henderson E. (1994). G-Wires: Self-assembly of a telomeric oligonucleotide, d(GGGTTGGG), into large superstructures. Biochemistry.

[50] Shi Y., Luo Q., Li N.B. (2013). A highly sensitive resonance Rayleigh scattering method to discriminate a parallel-stranded G-quadruplex from DNA with other topologies and structures. Chem. Commun..

